# Improving Gait Analysis Techniques with Markerless Pose Estimation Based on Smartphone Location

**DOI:** 10.3390/bioengineering11020141

**Published:** 2024-01-30

**Authors:** Junhyuk Yang, Kiwon Park

**Affiliations:** Department of Mechatronics Engineering, Incheon National University, Incheon 22012, Republic of Korea; jun_h@inu.ac.kr

**Keywords:** gait analysis, markerless, human pose estimation, kinematics, lower extremity

## Abstract

Marker-based 3D motion capture systems, widely used for gait analysis, are accurate but have disadvantages such as cost and accessibility. Whereas markerless pose estimation has emerged as a convenient and cost-effective alternative for gait analysis, challenges remain in achieving optimal accuracy. Given the limited research on the effects of camera location and orientation on data collection accuracy, this study investigates how camera placement affects gait assessment accuracy utilizing five smartphones. This study aimed to explore the differences in data collection accuracy between marker-based systems and pose estimation, as well as to assess the impact of camera location and orientation on accuracy in pose estimation. The results showed that the differences in joint angles between pose estimation and marker-based systems are below 5°, an acceptable level for gait analysis, with a strong correlation between the two datasets supporting the effectiveness of pose estimation in gait analysis. In addition, hip and knee angles were accurately measured at the front diagonal of the subject and ankle angle at the lateral side. This research highlights the significance of careful camera placement for reliable gait analysis using pose estimation, serving as a concise reference to guide future efforts in enhancing the quantitative accuracy of gait analysis.

## 1. Introduction

Accurate measurement and analysis of human gait plays a critical role in gaining insight into the intricate mechanics of human movement, identifying abnormal gait patterns, and developing effective treatments for gait-related disorders. To objectively analyze human gait, various kinematic and kinetic parameters such as stride length, walking speed, joint angles, and ground reaction forces are measured. These parameters serve as valuable indicators of gait quality, symmetry, and efficiency, providing a comprehensive assessment of an individual’s gait pattern [1,2,3,4,5,6].

In the past, observational gait analysis was the primary method for quantitatively evaluating joint movement during gait. However, this method is subjective, and the data obtained may not be entirely reliable [7]. To obtain more accurate and detailed gait analysis, reflective marker-based motion capture systems have been developed. In these systems, reflective markers are attached to the subject’s body and tracked with an infrared camera to obtain marker coordinates. Human motion is then analyzed using the coordinates of these markers. Marker-based 3D motion capture is a widely used measurement tool for accurately quantifying human motion parameters [8].

However, the marker-based 3D motion capture system is very expensive and requires specialized personnel to handle the equipment or operate the system. Due to the need for specialized equipment and controlled environments, it can be challenging to create a dedicated experimental space for analyzing human motion analysis. In addition, placing markers on the subject’s body for motion analysis is time-consuming. As a result, both people with disabilities and the general population may have difficulty accessing such equipment and conducting experiments in dedicated experimental spaces [9].

In recent years, markerless human pose estimation (HPE) systems have gained significant attention to overcome the limitations of marker-based motion capture systems. HPE is a system that uses machine learning and deep learning algorithms to detect human joint positions in images or videos. A large amount of image data is used to build a dataset that labels human joint key points, and this dataset is trained with a deep learning model to accurately detect human joint key points. In this process, a convolutional neural network (CNN), one of the deep learning models, is mainly used to train the datasets. A CNN model is a neural network composed of multiple layers, and input images are passed through this neural network. Features are extracted from images input through CNN, and these features are used to detect human joint key points [10]. The HPE system can automatically detect the position of human joints using only images recorded with a smartphone, making it easier, simpler, and cheaper to use for various applications such as motion analysis, gesture recognition, and detection. With the advancements in computing technology, the utilization of HPE systems is becoming increasingly widespread. In addition, the barriers to analyzing human motion using traditional motion capture methods are rapidly decreasing, leading to a growing body of research exploring the use of webcam or smartphone-based HPE systems [11,12,13,14,15,16,17,18,19].

Human pose estimation (HPE) systems can be broadly categorized into two types: 2D and 3D HPE. The 2D HPE system tracks human joints or body parts on the entire surface of an image or video, whereas the 3D HPE system estimates the depth of human joints or body parts in an image or video [20,21]. With the development of computing technology and the collection of large-scale datasets, HPE has made remarkable progress, particularly in 2D images and videos. The 3D HPE system, which provides depth information from images and videos, is also advancing, but its performance is not yet satisfactory, and there is ample room for improvement [22]. The 3D HPE system is particularly challenging due to various difficulties, such as depth ambiguity, complex background, lack of training data, occlusion, and scale deformation [23]. For gait analysis, 2D analysis in the side view can quantify motion and address specific clinical problems [24].

To date, there have been many studies using 2D HPE to analyze gait kinematics and temporal and spatial gait parameters in normal subjects or patients with gait disorders, and the broader topic of estimating gait variables or identifying gait features has been addressed. Lv et al. [25] analyzed the walking characteristics of patients with knee joint disorders using sample entropy fused with AlphaPose human pose estimation, and proposed an algorithm capable of discriminating between healthy individuals and those with knee joint disorders. Sato et al. [26] proposed a method using OpenPose to estimate cadence from frontal plane videos of Parkinson’s disease patients using sagittal and frontal plane gait video data from normal subjects. Stenum et al. [27] compared spatiotemporal and sagittal kinematic gait parameters measured with OpenPose to 3D motion capture and proposed a reliable analysis workflow to accurately estimate various gait parameters and detect changes in the gait patterns of healthy individuals. Viswakumar et al. [28] evaluated the performance of the markerless systems OpenPose and MS Kinect using lower extremity joint angle variables during gait and investigated the impact of different lighting and clothing conditions. Rohan et al. [29] proposed an efficient mechanism for gait analysis by combining human pose estimation and convolutional neural networks, achieving a 97.3% accuracy in classifying normal and abnormal gait. Takeda et al. [30] compared lower limb joint angles obtained through video analysis using OpenPose and Kinovea, a markerless motion capture method, with 3D motion capture data and suggested that OpenPose, one of the human pose estimators, could be a suitable substitute for 3D marker-based motion capture. Ota et al. [31] measured lower extremity joint angles during treadmill walking and running using OpenPose and Vicon and suggested that an OpenPose-based motion analysis system can measure the lower extremity range of motion in the sagittal plane. Hii et al. [32] proposed an automated method for temporal gait analysis using MediaPipe and validated this approach against the Vicon motion capture system, demonstrating excellent agreement in most temporal gait parameters.

Previous studies have focused on using 2D HPE systems to measure gait kinematics and temporal and spatial gait parameters, and compare them to 3D marker-based motion capture for effectiveness. In a study investigating technical issues of HPE when performing motion analysis using HPE, Viswakumar et al. [28] investigated the effects of clothing and ambient light intensity on gait analysis results using HPE. Takeichi et al. [33] investigated the effect of the frame rate of video recorded by a smartphone camera on running form analysis using HPE. However, the crucial aspect of camera placement for accurate calculation of gait variables using a single camera to investigate technical issues in HPE has often been overlooked. Recognizing these challenges, this paper addresses the issue by investigating the effect of camera positions during treadmill walking using five smartphone cameras.

Building on the existing body of knowledge, this study aimed to not only compare HPE with marker-based systems but also to enhance the performance and accuracy of HPE in gait analysis. The importance of minimizing occlusions and obtaining reliable joint information during walking has led to the investigation of the specific influence of camera positions on treadmill walking using five smartphone cameras. In contrast to previous research that addressed issues such as clothing effects and ambient light intensity, this research focused on refining the methodology by strategically placing cameras to mitigate challenges associated with leg occlusions during the gait cycle. Investigating the effect of camera placement on the joint detection rate of the HPE system will contribute to a broader understanding of the factors that influence the accuracy and efficiency of gait analysis in clinical and research settings. This effort has the potential to advance the current state of the field and pave the way for further research. By highlighting the importance of proper camera placement in 2D HPE-based gait analysis, this research aimed to fill a gap in the existing literature and explore methods to improve accuracy and efficiency in clinical and research settings.

## 2. Methods

### 2.1. Participants

A total of 20 healthy volunteers (10 males and 10 females; mean age: 24.15 ± 1.8 years; mean height: 169.4 ± 8.3 cm; mean mass: 64.93 ± 15.3 kg) from the normal population participated in this experiment. Individuals with a history of serious injuries such as musculoskeletal injuries, neurological disorders, fractures, or those who have undergone surgery were excluded. The participants provided written informed consent before the start of this study, and the Ethics Committee of the authors’ affiliated university approved the experimental procedure used in this study.

### 2.2. Data Collection

**Figure 1:**
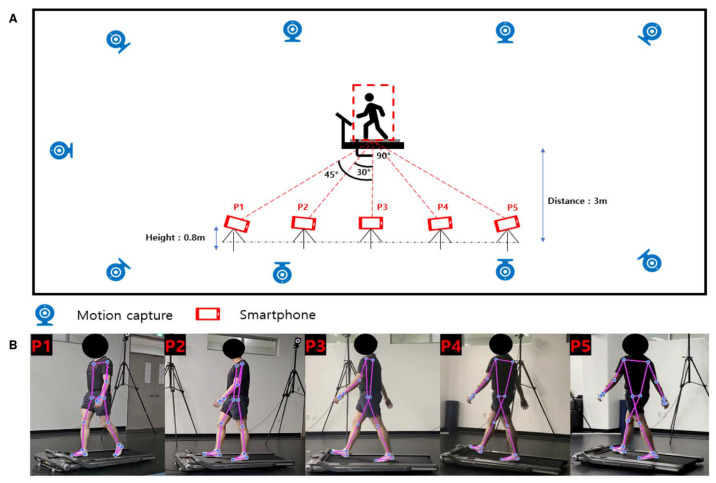
Overview of experimental setup. (**A**) 9 motion capture cameras and 5 smartphone cameras. The smartphone cameras are set at a distance of 3 m from the subject and a height of 0.8 m. (**B**) The skeleton model estimated by HPE according to each smartphone position.

Nine motion capture cameras (NaturalPoint, Corvallis, OR, USA, Optitrack Prime 41 Motion capture system, 4.1 MP, 120 fps) were used to measure gait motion. Each motion capture camera was strategically placed at an optimal distance to accurately capture the subject’s movements within the experimental space. The height of each infrared camera was standardized to 2.3 m to ensure consistent measurement conditions. A total of 39 reflective spherical markers were attached to anatomical landmarks for motion analysis: 4 on the head, 7 on each arm, 6 on each leg, 5 on the torso, and 4 on the pelvis. To prevent the markers from shaking at each joint attachment point, subjects were asked to wear shorts, short sleeves, or tight-fitting suits. Nevertheless, due to the possibility of distortion of the markers, we used Motive Optitrack software (version 3.0.2) to track the positions of the markers in real time, and manual editing is performed to minimize distortion. In addition, despite the participant wearing a T-shirt, we have implemented the preventive measures mentioned in the paper to ensure reliable data acquisition. Before this experiment, each subject adjusted the treadmill speed to their or her preferred speed, and a 3 min preliminary walking process was performed to adapt to walking on the treadmill. After the preliminary walking process, a 5 min break was taken, and each subject walked on the treadmill for 1 min at their preferred speed, and at the same time, the gait motion was recorded using 9 motion capture cameras and 5 smartphone cameras (Samsung Galaxy S22, 2340 × 1080 pixels, 30 fps). Preliminary experiments with smartphone cameras in front of and behind the subject showed that it was difficult to obtain reliable datasets for gait analysis. Since most gait analysis is performed from the side view, the experiment was conducted in five different positions to see if the most acceptable datasets could be obtained from a specific position in the side view. The smartphone cameras were positioned so that the angle between the subject and the smartphone was 90° from the subject’s side and 45° and 30°, respectively, (Figure 1). The distance between the smartphone camera and the subject was set to 3 m to ensure the subject’s whole body was included in the recording screen, and the height was set to 0.8 m. Based on several preliminary experiments, it was confirmed that distance and height did not critically affect the detection rate of pose estimation, as long as the subject’s whole body was captured on the camera screen.

### 2.3. Human Pose Estimation (HPE)

**Figure 2:**
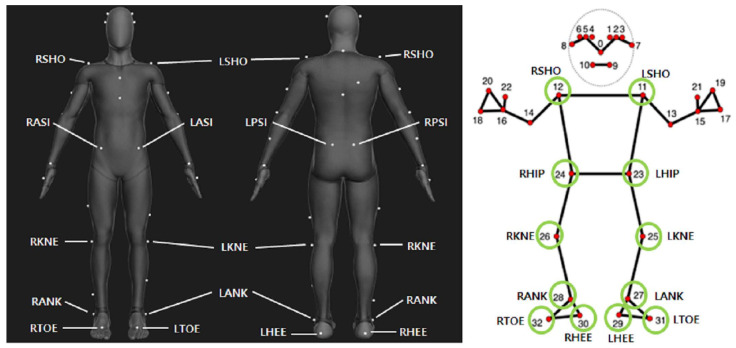
Comparison of marker positions between two devices required for kinematics gait analysis. The green circles represent the joints used to calculate the lower extremity joint angle among the 33 landmarks in Mediapipe.

Compared to other HPE open-source libraries, Mediapipe is relatively fast to process and shows relatively high accuracy in video-based analysis methods. In addition, the pipeline’s use of pose estimation for a single person makes it highly personalized for gait analysis [32,34,35,36]. Therefore, in this study, customized HPE based on Mediapipe (version 0.9.3), an open-source software provided by Google as a cross-platform pipeline processing tool [37], was utilized to perform gait analysis. Mediapipe is a real-time system that detects feature points in a single image captured using a digital camera and provides trained models for pose estimation of the face, hands, and body. BlazePose is the HPE model configured in CNN-based architecture, and it is Mediapipe’s pose detection model that can track human movement from 33 landmark key points. The pose of a person can be identified from the landmark key points(x-y-z coordinates) detected by this model. BlazePose combines an encoder–decoder network architecture, which predicts both heatmaps of the joints and the coordinates of the joints simultaneously. The initial pose alignment provided by the BlazePose detector is complemented by a tracker, effectively detecting key points, and the presence of a person, and refining the regions of interest. This sophisticated model was trained on a substantial dataset, comprising 60,000 images containing a single person or few people, and an additional 25,000 images with only a single person performing various exercises [38]. In this study, the gait motions of each subject were recorded with smartphone cameras, and the x, y coordinates of the shoulder, hip, knee, ankle, toe, and heel of the BlazePose model were used to perform 2D gait analysis (Figure 2).

### 2.4. Data Processing

Following the acquisition of motion capture data, marker data from the marker-based motion capture system was carefully examined using Motive Optitrack software for accurate detection and quality improvement. Editing procedures were performed to enhance data reliability, including gap-filling interpolation techniques to address missing marker data points or gaps in the motion capture sequence. The Motive Optitrack software’s interactive visualization tool was utilized to verify and adjust marker positions, minimizing inaccuracies caused by noise or tracking errors.

Simultaneously, videos taken by the smartphone camera underwent processing using the Mediapipe algorithm executed with Jupyter notebook. A total of 33 anatomical landmarks were extracted from each frame, and the resulting data were converted into a CSV file format containing landmark coordinates for each frame. To reduce jitter and noise effects, a moving average filter in MATLAB (version R2021 a) was applied to the coordinates of each joint obtained from Mediapipe.

**Figure 3:**
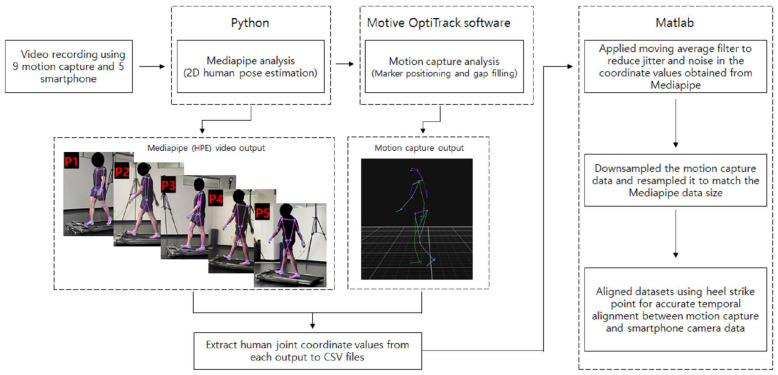
The workflow of the proposed approach.

To address the difference in frame rates between the two methods, motion capture data were downsampled and rescaled to match the frame rate of the smartphone camera using MATLAB. Any remaining temporal discrepancies between the two methods were treated with manual adjustments to the kinematic data to ensure the same time instant of heel strike as a reference point for initiation of gait cycle, with approximately 50 gait cycles of 1 min duration analyzed per participant (Figure 3).

**Figure 4:**
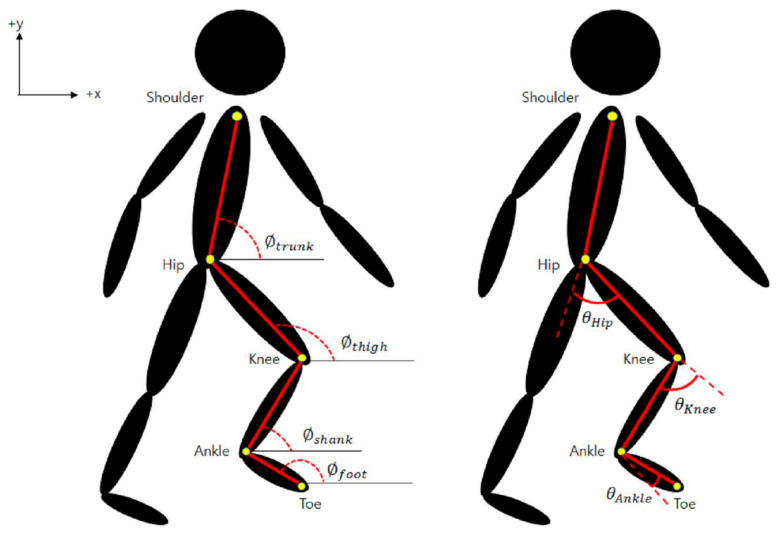
Representation of the kinematic model utilized for calculating hip, knee, and ankle joint angles.

The MATLAB software (version R2021 a) was used to import CSV files containing coordinate values for individual joints to facilitate gait analysis. Specifically, within the side view, the hip, knee, and ankle joint angles were calculated based on the coordinates of the shoulder, hip, knee, ankle, and toe joints from each CSV file. The gait cycle was determined by identifying the heel strike and toe-off points within the coordinate data. To calculate the joint angles, it was imperative to ascertain the dimensions of the trunk, thigh, shank, and foot segments. As shown in Figure 4, the following equations were used to obtain measurements for each segment and then calculate the corresponding joint angles, with joint angles calculated in degrees:

(1) $$\begin{array}{cc} & \phi_{segment}=tan^{-1}\left(\frac{\Delta y}{\Delta x}\right) \end{array}$$

(2) $$\begin{array}{cc} & \theta_{hip}=\phi_{thigh}-\phi_{trunk} \end{array}$$

(3) $$\begin{array}{cc} & \theta_{knee}=\phi_{thigh}-\phi_{shank} \end{array}$$

(4) $$\begin{array}{cc} & \theta_{ankle}=\phi_{foot}-\phi_{shank}-90^{{}^{\circ}} \end{array}$$

### 2.5. Error Calculation and Statistics

In this study, the reliability of the lower extremity joint angles obtained by the HPE system was evaluated using the mean absolute error (MAE), a frequently used metric when comparing kinematics between markerless and marker-based motion capture systems [27,31]. The MAE was calculated by subtracting the values obtained from the HPE system from those obtained by the marker-based motion capture system. The resulting differences were subjected to the absolute value function and divided by the total number of frames, thus considering only the magnitude of the errors. This approach allows a comprehensive evaluation of the overall reliability and effectiveness of the HPE system by comparing the results obtained from gait analysis using the HPE system with those obtained from the marker-based motion capture system. Lower MAE indicates a smaller difference in joint angles between the two methods, indicating the feasibility of using HPE in treadmill gait analysis. A higher correlation coefficient indicates a stronger association between the datasets, supporting the reliability of gait analysis using HPE.

(5) $$\begin{array}{cc} & Mean\;absolute\;error\left(Degree\right)=\frac{1}{N}\sum \left|Joint\;angle_{i}\left(M\right)-Joint\;angle_{i}\left(H\right)\right|\\ & i=1,2,\ldots ,N\;is\;the\;number\;of\;frame\\ & M:motion\;capture,\;H:human\;pose\;estimation \end{array}$$

The Pearson correlation coefficient was calculated to evaluate the relationship between lower extremity joint angles calculated from HPE data and lower extremity joint angles calculated from motion capture data.

(6) $$\begin{array}{cc} & \rho_{M,H}=corr\left(M,H\right)=\frac{cov(M,H)}{\sigma_{M}\sigma_{H}}\\ & cov:covariance,\;\sigma :standard\;deviations \end{array}$$

## 3. Results

**Figure 5:**
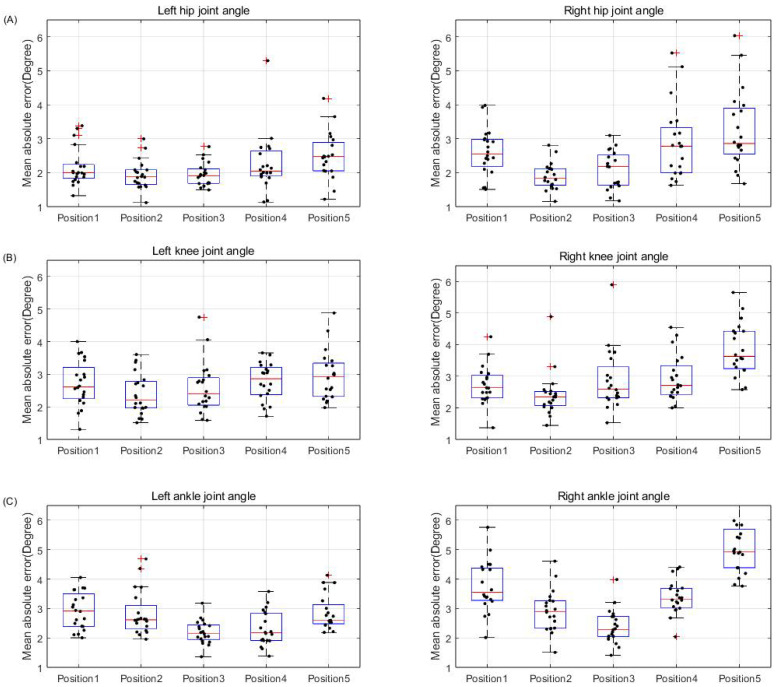
Boxplot of the MAE between motion capture and HPE for lower extremity joint angles. Each panel shows the MAE between HPE and motion capture for left and right lower extremity joint angles: (**A**) hip joint angle, (**B**) knee joint angle, (**C**) ankle joint angle. Boxplot shows the mean and SD of all subjects and individual subject data.

The majority of participants showed MAE of less than 5° between HPE and motion capture for hip, knee, and ankle joint angles (Table 1 and Figure 5). In addition, the correlation coefficient for lower extremity joint angles between HPE and motion capture was high, indicating that all joint angles were closely related to the true value (Table 2). In the case of hip joint angles, the lowest MAE of 1.92 ± 0.41 and high correlation coefficient of 0.97 ± 0.015 were observed at P2. Similarly, for knee joint angles, the lowest MAE of 2.4 ± 0.66 and the highest correlation coefficient of 0.98 ± 0.014 were observed at P2. For ankle joint angles, the lowest MAE of 2.29 ± 0.5 and the highest correlation coefficient of 0.84 ± 0.081 were observed at P3. Based on the average MAE and correlation coefficient of the subjects, the hip and knee joint angles were relatively accurately measured at P2, and the ankle joint angle was accurately measured at P3. Additionally, a difference was observed when comparing the left and right lower extremities. For hip joint angles, the average MAE on the left was 2.16 ± 0.63, whereas on the right, it was 2.54 ± 0.95. The average correlation coefficient was 0.96 ± 0.04 on the left and 0.94 ± 0.06 on the right. In the case of knee joint angles, the average MAE for the left was 2.68 ± 0.71, whereas for the right, it was 2.96 ± 0.9. The average correlation coefficients were 0.98 ± 0.01 on the left and 0.97 ± 0.02 on the right. As for ankle joint angles, the left exhibited an average MAE of 2.62 ± 0.66, whereas the right side had an MAE of 3.5 ± 1.17. The average correlation coefficients were 0.8 ± 0.11 on the left and 0.63 ± 0.2 on the right. Regarding the ankle joint angle, a notable observation was observed when comparing the results obtained from different camera positions. Specifically, at P5, most participants showed a relatively high MAE of 3.97 ± 1.35 and a comparatively low correlation coefficient of 0.63 ± 0.23.

## 4. Discussion

This study aimed to assess the reliability and effectiveness of the HPE system in gait analysis by comparing it with a marker-based motion capture system. When comparing previous gait analysis studies that used HPE with marker-based motion capture, Stenum et al. [27] reported MAE values of 4.0°, 5.6°, and 7.4° for hip, knee, and ankle joint angles, respectively. Viswakumar et al. [28] also reported MAE values of 7.73°, 5.82°, and 7.13° for hip, knee, and ankle joint angles between the two methods. In this study, the MAE values for hip, knee, and ankle joint angles were measured as 2.35°, 2.82°, and 3.06°, respectively. The results indicate a higher level of reliability compared to previous studies. However, it is important to note that our analysis was conducted on a treadmill, whereas the referenced studies were conducted in an overground walking setting. Therefore, it is crucial to consider the differences between these environments.

In addition, using five smartphone cameras, it was investigated whether the position of the smartphone camera affects the degree of joint detection associated with gait analysis results using the HPE system. The reliability and effectiveness of gait analysis using the HPE system were evaluated by calculating the MAE and correlation coefficient between the two methods based on hip, knee, and ankle joint angles. Overall, the results show that the MAE between HPE and marker-based motion capture for the lower extremity joint angles of most participants at all positions of the smartphone camera was less than 5° (Figure 5). This level of error is considered an acceptable margin of error in gait kinematic analysis [39]. The low MAE value was supported by a high correlation coefficient between HPE and marker-based motion capture for lower extremity joint angles (Table 2). Additionally, this study found that the position of the smartphone camera affected the average MAE and correlation coefficient.

**Figure 6:**
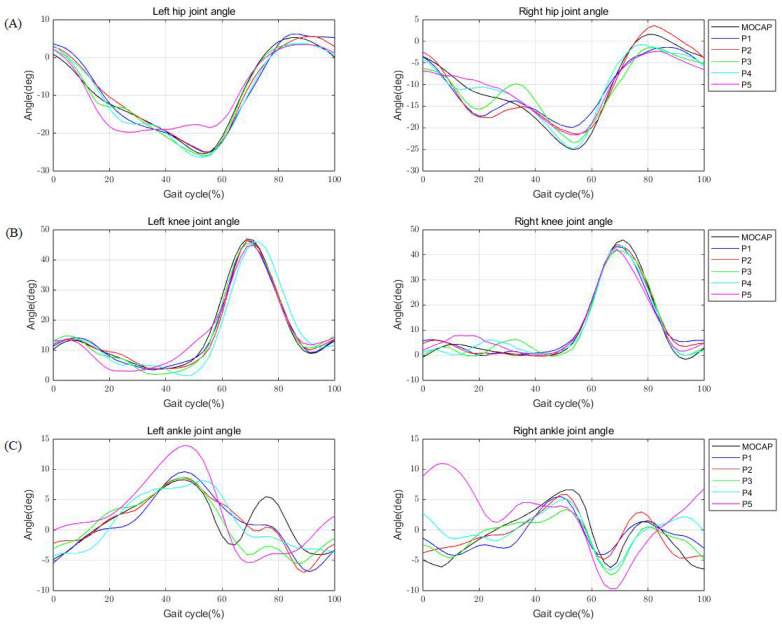
Example of one subject’s lower extremity joint angles between HPE and motion capture. Panels visually show the variation within the gait cycle to illustrate how the results of lower extremity joint angles using HPE for each smartphone position compare to motion capture. (**A**) Left and right hip joint angle, (**B**) knee joint angle, (**C**) ankle joint angle.

**Figure 7:**
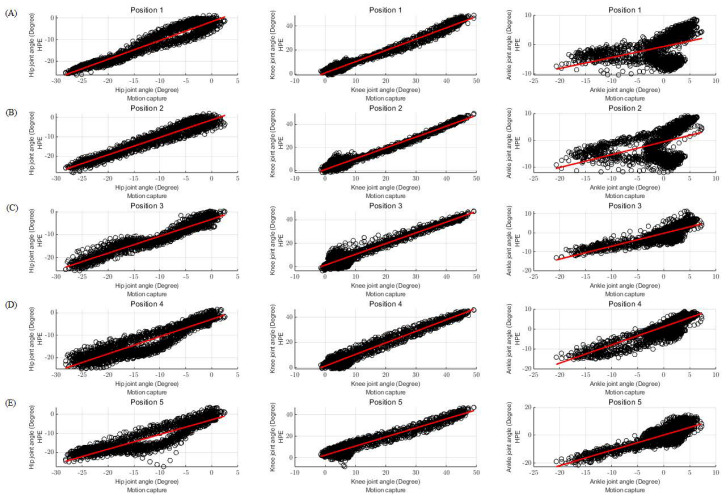
Example of left lower extremity joint angles comparisons among the different measurement systems. Panels show the relationship for lower extremity joint angles calculated using the two methods. (**A**) shows the relationship when the smartphone camera position is at P1, (**B**) at P2, (**C**) at P3, (**D**) at P4, and (**E**) at P5.

Examining the difference between the lower extremity joint angles of individual participants, which represent the most closely resembling data to the average dataset of all participants, in terms of HPE and motion capture, reveals that the differences in angles are not substantial, and similar trends can be identified (Figure 6). In addition, the values of the two methods were compared based on the lower extremity joint angles calculated using HPE and motion capture, and the relationship was confirmed (Figure 7). The results of the comparative study with motion capture demonstrate that the HPE approach is reliable and effective for treadmill gait analysis, as evidenced by the low MAE and high correlation coefficient. These results suggest the feasibility of using HPE for precise analysis of treadmill gait without requiring specialized equipment beyond a smartphone camera.

The MAE and correlation coefficient between motion capture and HPE for lower extremity joint angles vary depending on the position of the smartphone camera. Identifying the position where the lower extremity joint angle difference has the lowest MAE and high correlation coefficient implies that the respective joint angle is calculated most accurately at that position. The results of this study indicate that the hip and knee joint angles are most accurately calculated when the smartphone camera is positioned at P2, with the lowest MAE and high correlation coefficient. Similarly, the ankle joint angle is accurately calculated when the smartphone camera is positioned at P3, with the lowest MAE and the high correlation coefficient. At position P2, the detection of joints necessary for calculating hip and knee joint angles is superior compared to other positions, resulting in more accurate measurements. At position P3, the detection of joints relevant to ankle joint angle calculation surpasses other positions, leading to enhanced accuracy. The significance of specific camera positions in optimizing the precision of joint angle calculations using the HPE system is highlighted by this observation. These findings suggest that adjusting the position of the smartphone camera can enhance the performance of gait analysis using the HPE system. This adjustment addresses issues such as joint visibility, potential occlusions, and detection reliability, thereby improving the overall accuracy and effectiveness of gait analysis conducted with HPE systems. This highlights the critical role of optimal camera placement in refining gait analysis precision within HPE systems.

In addition, the MAE for the left lower extremity joint angles was relatively lower compared to the right, and the correlation coefficient was relatively higher. This means that the left lower extremity joint angle results are more reliable than the right lower extremity joint angle results from the gait analysis using HPE, suggesting that the right lower extremity joints are obscured by the left joints due to the positioning of the smartphone cameras on the left side of the subject. Based on these findings, it suggests that the positioning of the smartphone camera, whether on the left or right side of the participant, could potentially influence the results of gait analysis using HPE [27].

In the case of the ankle joint angle, the correlation coefficient appears to be relatively low compared to the consistently high coefficients observed for the hip and knee joint angles. Previous studies also showed results showing that the error in ankle joint angle was large or that the joint detection rate was relatively low in the area below the ankle [40,41,42]. Therefore, further investigation is necessary to identify the underlying factors contributing to this variance. A plausible explanation for this discrepancy lies in the intricate nature of ankle joint angle estimation within the HPE framework. Unlike the hip and knee joints, where key points are relatively more prominent and reliably detectable, the estimation of the ankle joint angle heavily relies on accurate detection of the toe key points. The complexity of toe detection, which can be susceptible to occlusion and variability in footwear, might introduce inherent challenges that impact the precision of ankle joint angle calculations. Excluding P3, the correlation coefficients for the right ankle joint at the remaining positions are relatively low. This indicates that toe detection for calculating ankle joint angles was not accurately performed, particularly during the crossover of the left and right legs when the right toe was obscured by the left lower limb joint, resulting in suboptimal joint detection rates. In particular, the correlation coefficient for the right ankle joint angle at P5 was the lowest at 0.45 ± 0.149. This suggests that when the camera is positioned behind the subject for HPE to calculate joint angles, the toe of the right ankle is often obscured or inaccurately detected by other lower limb joints. Nevertheless, despite the challenges at P5, the higher correlation coefficient for the left ankle joint angle compared to the right indicates that, due to the camera being placed on the left side of the subject, the detection of the left toe was more accurate than that of the right toe. Given these findings, it becomes evident that prudent caution is essential when relying solely on ankle joint angle measurements derived from HPE. This is especially important in scenarios where accurate ankle joint angle information is critical, such as in evaluating specific pathologies or athletic performance. The observed limitation emphasizes the necessity for additional research and improvement of techniques for estimating ankle joint angles in the wider context of gait analysis based on HPE.

Nevertheless, for most subjects, the MAE between motion capture and HPE for all joint angles tend to be less than 5°, which is an acceptable error range for gait kinematics analysis, and the correlation coefficients also show high values [39]. These results indicate that the use of HPE provides joint detection results that closely resemble the actual joints detected using motion capture. These results are significant in both academic and clinical contexts. They highlight the capability of HPE to produce joint detection results that closely mirror the actual joints identified by motion capture methods. This alignment emphasizes the feasibility of utilizing HPE for treadmill-based gait analysis. The use of a smartphone camera as the primary device is a notable advantage, eliminating the need for specialized machinery. The implications of this extend beyond convenience, catering to individuals with mobility limitations, as well as paving the way for gait analysis to be performed in domestic settings, increasing accessibility to the general population.

This study addresses a notable research gap by examining the impact of smartphone camera positions on the accuracy of joint detection in 2D HPE-based gait analysis. This gap is crucial as it pertains to the practical implementation of such systems in real-world scenarios. Through a thorough evaluation of various camera positions, we aim to offer valuable insights for enhancing the utilization of HPE systems in gait analysis, a crucial aspect of biomechanics and clinical assessments. The significance of our findings extends to settings where accommodating specialized equipment may be challenging. In contexts where dedicated gait analysis facilities or advanced motion capture systems are not feasible, our study suggests that 2D HPE-based methods, informed by the insights gained from diverse smartphone camera positions, can provide a reliable alternative. This study enhances the reliability and applicability of 2D HPE-based methods in the field of gait analysis and biomechanics, offering practical implications for researchers and practitioners in both well-equipped and resource-constrained environments.

## 5. Limitations

It is imperative to recognize the influence of various environmental and technological factors on the accuracy and consistency of HPE results. These include fluctuations in lighting conditions, variations in ambient light intensity, differences in the texture and color of subjects’ clothing, and even factors such as frames per second and the performance capabilities of the smartphone camera performance. These complex variables underscore the need to recognize the limitations and potential sources of variability inherent in the HPE process. The interplay of these factors highlights the importance of cautious interpretation of results, particularly in scenarios where precision is paramount. In light of these considerations, future research efforts could focus on understanding the intricate interplay between environmental factors, technological specifications, and resultant HPE accuracy. Such a comprehensive investigation has the potential to refine the application of HPE and enhance its utility in academic, clinical, and broader contexts. Furthermore, it could contribute to the establishment of standardized protocols for optimized HPE-based gait analysis.

Our study is limited by the fact that we primarily focused on estimating flexion–extension (2D) during treadmill walking, and whereas this provides valuable insights, it is crucial to acknowledge that other joint movements and additional planes of motion, particularly relevant in clinical applications, were not explored in this study. Future research should address these limitations by expanding the scope to include a broader range of joint motions and dimensions.

Although our reported results indicate that the differences in joint angles between pose estimation and a marker-based system are below the commonly accepted threshold of 5° for gait analysis [39], it is imperative to recognize the potential significance of this discrepancy. A 5° difference, although deemed acceptable in general gait analysis, may be non-negligible in certain kinematic or biomechanical assessments where precision is critical. Researchers and practitioners should exercise caution when applying our findings in contexts where higher accuracy is required.

This study specifically analyzed lower extremity joint angles during treadmill walking. To gain a more comprehensive understanding of the influence of camera positions, it is necessary to extend our investigation to overground walking scenarios. Additionally, exploring the impact of camera positions on temporal walking variables would further contribute to the field’s knowledge. Future research endeavors should consider these aspects to provide a more holistic view of the relationship between camera placement and gait analysis outcomes.

## 6. Conclusions

This study presents the effectiveness of a customized human pose estimation (HPE) system in analyzing treadmill gait, which is compared with marker-based motion capture. The analysis of lower extremity joint angles shows an acceptable level of MAE below 5° and high correlation coefficients between HPE and motion capture. It is also important to note that the accuracy of the HPE system is affected by the position of the smartphone camera, considering that the hip and knee joint angles are accurately calculated at P2 and the ankle joint angle is accurately calculated at P3. This study highlights the potential of HPE adoption in various fields, such as rehabilitation and sports science. Future research will investigate the factors that affect HPE accuracy and establish standardized protocols, contributing to advancements in biomechanics.

## Figures and Tables

**Table 1:** MAE between motion capture and HPE for lower extremity joint angles.

Joint Angles		Number of Subjects	Mean ± SD					*p*
			**P1**	**P2**	**P3**	**P4**	**P5**	
Hip (°)	Left	20	2.16 ± 0.56	1.94 ± 0.42	1.95 ± 0.34	2.26 ± 0.86	2.51 ± 0.69	0.018
	Right	20	2.61 ± 0.69	1.9 ± 0.4	2.07 ± 0.55	2.87 ± 1.09	3.26 ± 1.14	<0.001
Knee (°)	Left	20	2.71 ± 0.7	2.39 ± 0.64	2.58 ± 0.79	2.78 ± 0.57	2.96 ± 0.76	0.042
	Right	20	2.72 ± 0.61	2.41 ± 0.7	2.87 ± 0.96	2.94 ± 0.72	3.85 ± 0.83	<0.001
Ankle (°)	Left	20	2.92 ± 0.62	2.83 ± 0.75	2.19 ± 0.4	2.32 ± 0.59	2.85 ± 0.59	<0.001
	Right	20	3.74 ± 0.86	2.91 ± 0.71	2.39 ± 0.58	3.38 ± 0.57	5.09 ± 0.89	<0.001

**Table 2:** Correlation coefficient between motion capture and HPE for lower extremity joint angles.

Joint Angles		Number of Subjects	Mean ± SD				
			**P1**	**P2**	**P3**	**P4**	**P5**
Hip	Left	20	0.96 ± 0.017	0.97 ± 0.014	0.97 ± 0.023	0.94 ± 0.077	0.94 ± 0.044
	Right	20	0.95 ± 0.03	0.96 ± 0.016	0.96 ± 0.026	0.91 ± 0.114	0.92 ± 0.04
Knee	Left	20	0.98 ± 0.014	0.98 ± 0.017	0.98 ± 0.018	0.98 ± 0.011	0.98 ± 0.011
	Right	20	0.98 ± 0.01	0.98 ± 0.012	0.97 ± 0.025	0.97 ± 0.02	0.96 ± 0.021
Ankle	Left	20	0.73 ± 0.124	0.75 ± 0.113	0.84 ± 0.074	0.84 ± 0.095	0.81 ± 0.124
	Right	20	0.53 ± 0.18	0.7 ± 0.194	0.83 ± 0.087	0.67 ± 0.1	0.45 ± 0.149

## Data Availability

Data are contained within the article.
